# The FASILA Score: A Novel Bio-Clinical Score to Predict Massive Blood Transfusion in Patients with Abdominal Trauma

**DOI:** 10.1007/s00268-019-05289-0

**Published:** 2019-11-20

**Authors:** Ayman El-Menyar, Husham Abdelrahman, Hassan Al-Thani, Ahammed Mekkodathil, Rajvir Singh, Sandro Rizoli

**Affiliations:** 1 Department of Surgery, Clinical Research, Trauma and Vascular Surgery, Hamad General Hospital, P.O Box 3050, Doha, Qatar; 2 Department of Clinical Medicine, Weill Cornell Medical College, Doha, Qatar; 3 Department of Surgery, Trauma Surgery, Hamad General Hospital, Doha, Qatar; 4 Department of Surgery, Biostatistician, Hamad General Hospital, Doha, Qatar

## Abstract

**Background:**

Early identification of patients who may need massive blood transfusion remains a major challenge in trauma care. This study proposed a novel and easy-to-calculate prediction score using clinical and point of care laboratory findings in patients with abdominal trauma (AT).

**Methods:**

Patients with AT admitted to a trauma center in Qatar between 2014 and 2017 were retrospectively analyzed. The FASILA score was proposed and calculated using focused assessment with sonography in trauma (0 = negative, 1 = positive), Shock Index (SI) (0 = 0.50–0.69, 1 = 0.70–0.79, 2 = 0.80–0.89, and 3 ≥ 0.90), and initial serum lactate (0 ≤ 2.0, 1 = 2.0–4.0, and 2 ≥ 4.0 mmol/l). Outcome variables included mortality, laparotomy, and massive blood transfusion (MT). FASILA was compared to other prediction scores using receiver operating characteristics and areas under the curves. Bootstrap procedure was employed for internal validation.

**Results:**

In 1199 patients with a mean age of 31 ± 13.5 years, MT, MT protocol (MTP) activation, exploratory laparotomy (ExLap), and hospital mortality were related linearly with the FASILA score, Injury Severity Score, and total length of hospital stay. Initial hemoglobin, Revised Trauma Score (RTS), and Trauma Injury Severity Score (TRISS) were inversely proportional. FASILA scores correlated significantly with the Assessment of Blood Consumption (ABC) (*r* = 0.65), Revised Assessment of Bleeding and Transfusion (RABT) (*r* = 0.63), SI (*r* = 0.72), RTS (*r* = − 0.34), and Glasgow Coma Scale (*r* = − 0.32) and outperformed other predictive systems (RABT, ABC, and SI) in predicting MT, MTP, ExLap, and mortality.

**Conclusions:**

The novel FASILA score performs well in patients with abdominal trauma and offers advantages over other scores.

**Electronic supplementary material:**

The online version of this article (10.1007/s00268-019-05289-0) contains supplementary material, which is available to authorized users.

## Introduction

Uncontrolled bleeding is the leading preventable cause of death from trauma worldwide. Nearly half of all deaths within the first 24 h after trauma are caused by exsanguination and coagulopathy [1]. Massive transfusion (MT), defined as the transfusion of 10 or more units of packed red blood cells (PRBCs) in 24 h [1, 2], is required in 3% of patients with trauma. MT is usually unplanned and requires large quantities of blood; however, it is often the differentiating factor between life and death [1]. Delays in activating massive transfusion protocols (MTP) may adversely impact patients’ outcomes, while inappropriate activation may waste resources and incur costs to the institutions. Timely, sustained, and appropriate MTP activation remains a challenge to all trauma centers and the cost-effectiveness process [3–6].

MTP activation relies heavily on the subjective clinical judgment of initial vital signs and on the response to initial resuscitation. Various scoring systems have been proposed to identify patients in need of MT. Currently, there are nearly two dozen military and civilian prediction scores in the medical literature. However, no universal consensus has been reached, and none of them have been widely adopted [2–4, 7–22]. Many scores use time-consuming laboratory tests along with physiologic and anatomical parameters [2–4, 7, 9–12, 14, 20], whereas others use physiologic parameters along with point of care (POC) tests [8, 15, 17–19]. However, few of these scores are simple, efficient, and easy to remember and coincidentally are the ones most commonly used in practice. They include the Assessment of Blood Consumption (ABC) score, mostly for penetrating trauma, the Shock Index (heart rate/systolic blood pressure), and the Revised Assessment of Bleeding and Transfusion (RABT) score [8]. However, these scores have several limitations.

Our group proposes the “FASILA score,” which combines clinical, physiological, and laboratory parameters that are individually reliable predictors of mortality and the need for blood transfusion. We hypothesized that the incorporation of focused assessment with sonography in trauma (FAST), SI, and serum lactate into one scoring tool (the FASILA score) would provide an accurate, simple, and easy-to-remember scoring system, offering superior outcomes compared to other prediction scores. FAST is a routine, primary adjuvant test to detect blood in the peritoneum in abdominal trauma. FAST positivity or negativity, and/or the number of positive regions, are well-known determinants of MT requirements [3, 8, 9, 23]. However, the accuracy of FAST depends on the technical skill of the operator and on patient-related factors including obesity.

The heart rate (HR) and systolic blood pressure (SBP) are universally employed for the initial evaluation of injured patients and have been included in several prediction models [3, 7–11, 16, 20]. The HR/SBP ratio, referred to as the Shock Index (SI), outperforms HR or SBP alone in predicting MT [22, 24]. However, HR and SBP have significant limitations and are affected not only by bleeding, but also by timing, anxiety, stress, and medications.

Serum lactate has been used as a diagnostic and prognostic parameter for hemorrhagic shock for many decades. However, few prediction models have incorporated it [10, 23]. Sohn et al. [25] recently reported that combining initial lactate with SI improves the predictive performance for MT in primary postpartum hemorrhage. Despite its limitations, serum lactate levels correlate well with shock, mortality, and response to resuscitation efforts. Although it is relatively expensive and not available in many trauma centers, introducing the point of care (POC) to measure serum lactate accelerates the laboratory process to get its results in few seconds with subsequent early decision.

Prediction models perform better than clinical judgment alone [26]. However, the existence of dozens of scoring models indicates their inadequacies and reflects the heterogeneous and contrasting approaches to decision-making in early trauma resuscitation [26]. We speculate, in addition to accuracy, simplicity of calculation (as in the FASILA score) is of prime importance for an ideal prediction score in early trauma resuscitation. The aim of the current study was to introduce and test the utility of this novel simple score (FASILA) using clinical and POC laboratory findings, to predict MT and mortality in patients with abdominal trauma, and to compare it with the other widely used scores.

## Methods

Data were obtained from the prospectively collected Qatar National Trauma Registry for all patients with abdominal trauma who were admitted to the Hamad Trauma Center (HTC) between 2014 and 2017 and were retrospectively analyzed. All patients with documented FAST results, initial vital signs (SBP and HR), and initial serum lactate were included. We excluded patients with pre-hospital cardiac arrest. The primary outcome of the study was the requirement of MT (i.e., transfusion of 10 units or more of PRBCs within the first 24 h of trauma). This study focused on patients with abdominal injury because the abdomen is an important and frequent site of bleeding in cases of trauma that requires hemostatic resuscitation (including MT), surgery, and other hemostatic interventions. In this study, abdominal trauma was defined based on the ICD-9 (code 863-869).

The baseline and clinical characteristics of all patients, including age, type of trauma (blunt or penetrating), initial vital signs, initial laboratory findings, quantity of blood transfusion, length of hospital stay, intensive care unit (ICU) admission, number of days on the ventilator, and in-hospital deaths, were retrieved from the electronic medical records. Initial vital signs in the emergency department (ED) including SBP, diastolic BP, HR, and oxygen saturation were obtained.

The FASILA score is the sum of the following parameters: FAST tests (negative = 0, positive = 1), SI (0 = 0.50–0.69, 1 = 0.70–0.79, 2 = 0.80–0.89, and 3 ≥ 0.90), and initial serum lactate (0 ≤ 2.0, 1 = 2.0–4.0, and 2 ≥ 4.0 mmol/l). The minimum and maximum scores were 0 and 6, respectively.

Serum lactate was estimated on arrival using POC testing (ABL90 FLEX blood gas analyzer), which delivers 17 parameters within 35 s from as little as 65 μL of blood [27]. The normal range of SI in healthy adults is between 0.5 and 0.7 [25]. Initial pulse pressure was defined as the difference between SBP and DBP at the ED.

The ABC score is the sum of FAST (positive = 1), SBP (≤ 90 mmHg = 1), HR (≥ 120 bpm = 1), and mechanism of injury (MOI) (penetrating = 1) [24].

The RABT score includes the FAST result (positive = 1), SI (> 1 = 1), pelvic fracture (present = 1), and MOI (penetrating = 1) [28].

The Revised Trauma Score (RTS 0–12) comprises three parameters, namely, the Glasgow Coma Scale (GCS), SBP, and respiratory rate.

The Trauma and Injury Severity Score (TRISS) is a combination index based on the RTS, Injury Severity Score (ISS), and patient's age [29].

The Hamad Trauma Center is the only tertiary level 1 national trauma center in Qatar. It provides treatment for moderate to severe traumatic injuries. Emergency treatment is freely accessible to everyone living in Qatar. Qatar (approximately 2.6 million population) has a mature and well-established trauma system, which was the first trauma organization in the world accredited by the Accreditation Canada International (ACI), attesting to the high quality and safety of the care provided. The HTC receives approximately 2500 patients with traumatic injuries per year (approximately 1500–2000 patients require hospital admission annually); the majority (45%) have road traffic injuries (RTI). Abdominal trauma accounts for approximately 15% of all trauma-related admissions, of which approximately 2/3 are RTI cases [30].

This study was conducted in accordance with the institutional ethical standards and after approval from the Research Ethics Committee of the Medical Research Center, Hamad Medical Corporation (IRB # MRC-01-18-003). A waiver of consent was granted as there was no direct contact with patients, and the data were anonymously collected. This study included the STROBE checklist (Supplementary Table 1).

### Statistical analysis

Data were presented as means ± standard deviations (SD), medians (range/interquartile range) and 95% confidence intervals as appropriate, for continuous variables, and as frequencies and proportions for categorical variables. A comparison was made between patients who received MT and those who did not.

Receiver operating characteristic (ROC) curve analysis was performed for the optimum FASILA cutoff score, plotted against blood transfusion. Patients were divided into 2 groups based on the FASILA cutoff value (low vs. high score groups), and differences between the groups were analyzed. The area under the curve (AUC) and the c-statistic were calculated to evaluate the performance and discriminatory power of the FASILA score. The sensitivity, specificity, positive predictive value (PPV), and negative predictive value (NPV) of the score in predicting the need for MT were determined. Furthermore, FASILA score was categorized into 7 points from 0 to 6, and the 7-point FASILA scales were analyzed and compared. Differences in categorical variables between the respective comparison groups were analyzed using either the Chi-square or Fisher’s exact tests. Continuous variables were analyzed using either the Student’s *t* or analysis of variance (ANOVA) tests. Correlation coefficients were used to measure the strength of the relationship between the FASILA score and ISS, TRISS, RTS, GCS, RABT, ABC, and quantity of blood transfused.

A two-sided *p* < 0.05 was considered statistically significant. The bootstrap procedure was used for internal validation. We used bootstrap, sampling with replacement from the original data, which is a technique to predict the fit of a model to a hypothetical testing set when an explicit data or temporal data set is not available [31]. Logistic regression on 200 samples with replacement using simple random sampling from original data set was used to see bias and 95% confidence interval of percentile type for FASILA score. All statistical analyses were performed using Statistical Package for the Social Sciences (SPSS) for Windows version 21.0 (SPSS Inc; Chicago, IL, USA).

## Results

**Fig. 1:**
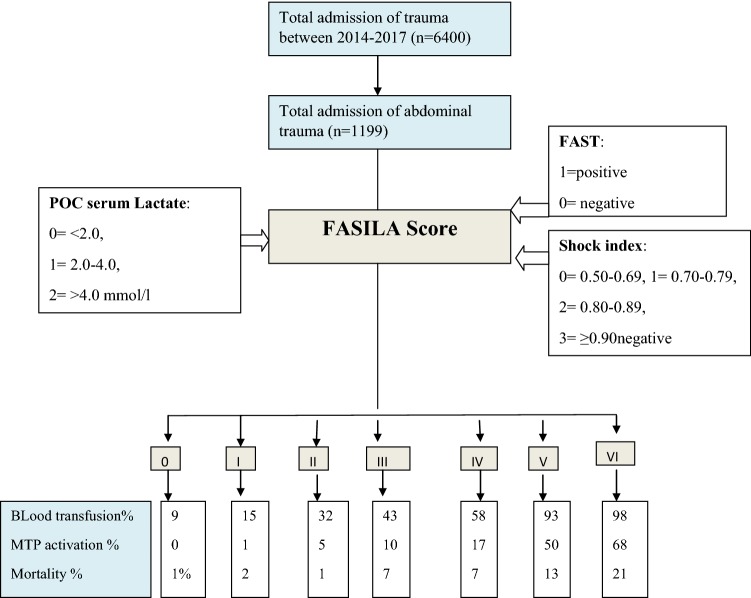
The study design and outcomes

Approximately 6400 injured patients were admitted to the Hamad Trauma Center between 2014 and 2017. Among them, 1199 patients with abdominal trauma were included in this analysis. The majority (*n* = 1111; 93%) suffered blunt trauma, and 90% were male. The mean age of the cohort was 31 ± 13.5 years. The most prevalent mechanism of injury was RTI (60%), followed by fall from heights (20%). Blood transfusion (any amount) was necessary in 477 patients (40% of the cohort); of them MTP was activated in 170 patients, whereas 138 received ≥ 10 units of PRBC in 24 h. Exploratory laparotomy was performed in 27% (*n* = 326) of the patients. FAST scan was positive in 30% and negative in 70% of cases. The overall median FASILA score was 3 (0–6); the score was lower in cases of penetrating trauma [2 (0–6)], compared with blunt trauma [3 (0–6)] (*p* = 0.73) and was higher in the pediatric group [4 (0–6)], compared with the adult group [3 (0–6)] (*p* = 0.002). Figure 1 shows the study design and outcomes.

Supplementary Table 2 shows the differences between the characteristics and outcomes of patients who received MT, compared to those who did not. Patients who received MT had higher FASILA scores, higher ISS, a higher incidence of laparotomy, longer hospital stay, and higher mortality (*p* = 0.001).

Validation of predictive models showed that 0.003 bias with 0.09 standard error in the model for the coefficient of FASILA (0.65; 95% CI 0.475–0.854; *p* = 0.005), i.e., 0.003/0.65 × 100 = only 0.5% biasness in the coefficient suggesting appropriateness for generalizing the model.

Table 1 shows the relationship between the 7-point FASILA scores and patient characteristics, laboratory findings, outcomes, and other injury scores (ISS, RTS, TRISS, and ABC scores). The initial hemoglobin levels decreased with increases in the FASILA score, while the white cell count and serum lactate levels increased exponentially (*p* = 0.001). Similarly, SI progressed linearly and was > 1.0 when the FASILA score was ≥ 4.0. When the FASILA score was high, i.e., between 4 and 6 (19–87%), there was a significant increase in the proportion of patients with ABC scores ≥ 2. The median ISS increased significantly, particularly for FASILA scores between 4 and 6; in contrast, the TRISS and RTS were inversely proportional (*p* = 0.001).

**Table 1:** Patients characteristics, laboratory findings, injury scores, and hospital course based on the FASILA score

Variable	Score 0	Score 1	Score 2	Score 3	Score 4	Score 5	Score 6	*p*
Age	35.43 (32.68–38.18)	34.04 (32.43–35.65)	33.32 (31.58–35.06)	32.90 (31.0–34.8)	32.27 (30.18–34.36)	32.68 (30.58–34.78)	33.62 (30.58–36.66)	0.477
Initial HB	14.05 (13.72–14.38)	14.13 (13.87–14.38)	14.75 (13.13–16.35)	13.32 (12.92–13.71)	12.96 (12.56–13.35)	12.18 (11.75–12.60)	11.59 (10.86–12.32)	0.001
Initial WBC	12.84 (11.68–13.99)	15.0 (14.09–15.91)	16.11 (15.12–17.11)	15.95 (14.83–17.07)	16.76 (15.62–17.89)	18.64 (17.19–20.09)	16.01 (14.05–17.98)	0.001
Serum lactate	1.42 (1.34–1.49)	2.22 (2.10–2.34)	2.83 (2.56–3.09)	4.71 (1.37–8.05)	3.18 (2.96–3.40)	4.56 (4.18–4.95)	6.98 (6.03–7.94)	0.001
Shock Index	0.59 (0.58–0.61)	0.65 (0.63–0.66)	0.70 (0.69–0.72)	0.83 (0.81–0.85)	1.02 (0.97–1.07)	1.29 (1.21–1.37)	1.33 (1.20–1.46)	0.001
Pulse pressure	51 ± 12	48 ± 14	46 ± 11	42 ± 15	40 ± 11	35 ± 11	34 ± 13	0.001
Scene O_2_ saturation	98 ± 2	97 ± 33	96 ± 4	95 ± 12	94 ± 10	92 ± 12	89 ± 20	0.001
ABC score ≥ (2%)	0	1.7(0.23–3.70)	5.4(1.73–9.15)	7.0(2.52–11.43)	18.9(12.00–25.80)	50(40.32–59.69)	87(77.33–97.40)	0.001
TRISS	0.98 (0.97–0.99)	0.98 (0.97–0.99)	0.96 (0.94–0.98)	0.93 (0.90–0.95)	0.91 (0.86–0.95)	0.86 (0.81–0.90)	0.79 (0.70–0.89)	0.001
RTS	7.75 (7.6–7.9)	7.74 (7.65–7.82)	7.53 (7.37–7.69)	7.2 (6.96–7.45)	7.08 (6.82–7.34)	6.69 (6.36–7.02)	6.4 (5.89–6.96)	0.001
ISS^a^	12(5–17)	13(8–17)	14(9–24)	17(9–23)	17(12–29)	27(17–34)	27(17–37)	0.001
Blood amount	2.88 (1.43–4.32)	4.64 (2.37–6.91)	4.53 (3.24–5.83)	6.71 (4.58–8.85)	7.08 (5.89–8.28)	11.62 (9.32–13.92)	13.15 (9.19–17.11)	0.001
Ventilator days	5.6 (1.0–11.91)	10.29 (5.09–15.48)	8.15 (5.06–11.25)	8.15 (5.92–10.37)	9.31 (6.67–11.95)	10.04 (7.71–12.37)	7.26 (5.15–9.38)	0.649
ICU LOS	3.95 (1.16–6.74)	7.14 (4.24–10.05)	8.36 (5.9–10.81)	9.64 (6.64–12.64)	12.16 (9.3–15.02)	14.4 (10.29–18.51)	13.02 (9.35–16.70)	0.005
Total HLOS	7.02 (5.25–8.79)	10.57 (8.01–13.14)	14.14 (11.57–16.72)	17.04 (13.26–20.82)	23.31 (14.91–31.72)	34.03 (27.07–40.99)	26.11 (19.75–32.46)	0.001

Data presented as mean and 95% confidence interval

*HB* hemoglobin, *WBC* white blood cell count, *ISS* Injury Severity Score, *TRISS* Trauma and Injury Severity Score, *RTS* Revised Trauma Score, *LOS* length of stay, *H* hospital, *ICU* intensive critical care

^a^Median and IQR

The ROC curve showed that the optimum FASILA score associated with blood transfusion was 4.5 (18.7% of the cohort had higher score). Supplementary table 3 shows the comparison between FASILA scores of < 4.5 vs. ≥ 4.5. Compared to FASILA scores of < 4.5, higher FASILA scores were associated with higher ISS, MT, MTP activation, laparotomy, longer hospital stay, and mortality. The optimum FASILA cutoff (4.5) was determined by the ROC curve with AUC 0.81(0.78–0.84); *p* = 0.001 (suppl Fig. 1) with 97% specificity and 90% positive predictive value.

### Correlation coefficients

Table 2 shows the significant correlations between the FASILA score and the other parameters/scores. It demonstrates that the FASILA score is directly related to RABT (*r* = 0.63), ABC (*r* = 0.65), and ISS scores (*r* = 0.39) and is inversely related to TRISS (*r* = − 0.30), RTS (*r* = − 0.34), scene oxygen saturation (*r* = − 0.21), GCS (*r* = − 0.32), and pulse pressure (*r* = − 0.37); (*p* = 0.001 for each variable).

**Table 2:** Pearson correlation coefficient analysis for FASILA score

ABC score	Pearson correlation (*r*)	0.655
	Sig. (2-tailed)	0.001
	*N*	820
RABT	Pearson correlation (*r*)	0.634
	Sig. (2-tailed)	0.001
	*N*	954
Injury Severity Scoring	Pearson correlation (*r*)	0.386
	Sig. (2-tailed)	0.001
	*N*	816
TRISS	Pearson correlation (*r*)	− 0.303
	Sig. (2-tailed)	0.001
	*N*	725
Revised Trauma Scoring	Pearson correlation (*r*)	− 0.340
	Sig. (2-tailed)	0.001
	*N*	728
Shock Index	Pearson correlation (*r*)	0.718
	Sig. (2-tailed)	0.001
	*N*	820
Blood amount	Pearson correlation (*r*)	0.321
	Sig. (2-tailed)	0.001
	*N*	353
Glasgow Coma Scale at ED	Pearson correlation (*r*)	− 0.320
	Sig. (2-tailed)	0.001
	*N*	800
Scene SPo2	Pearson correlation (*r*)	− 0.21
	Sig. (2-tailed)	0.001
	*N*	706
Pulse pressure	Pearson correlation (*r*)	− 0.37
	Sig. (2-tailed)	0.001
	*N*	920
Age > 14 years^a^	Pearson correlation (*r*)	− 0.0.08
	Sig. (2-tailed)	0.02
	*N*	875

^a^For age < 14 years old (*n* = 63): *r* = − 0.15; *p* 0.23

**Fig. 2:**
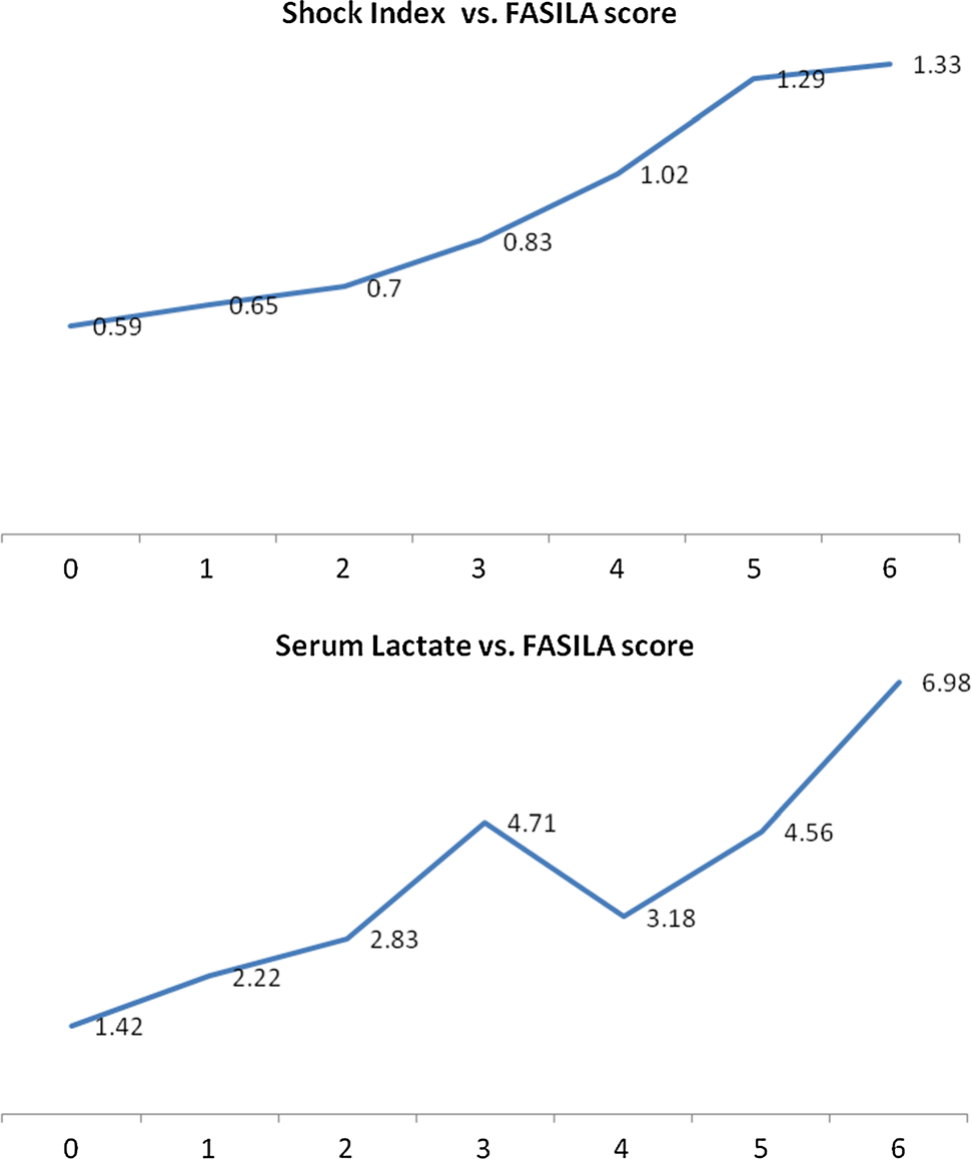
FASILA scores against Shock Index (upper) and serum lactate (lower panel)

Figure 2 shows the association between the FASILA score, serum lactate, and SI; the linear relationship between SI and the FASILA score is evident. In this cohort, serum lactate reached its peak (6.98 mmol/l) at a FASILA score of 6, while scores of 0 and 1 were associated with normal serum lactate levels (1.42–2.22 mmol/l).

### Outcomes

**Fig. 3:**
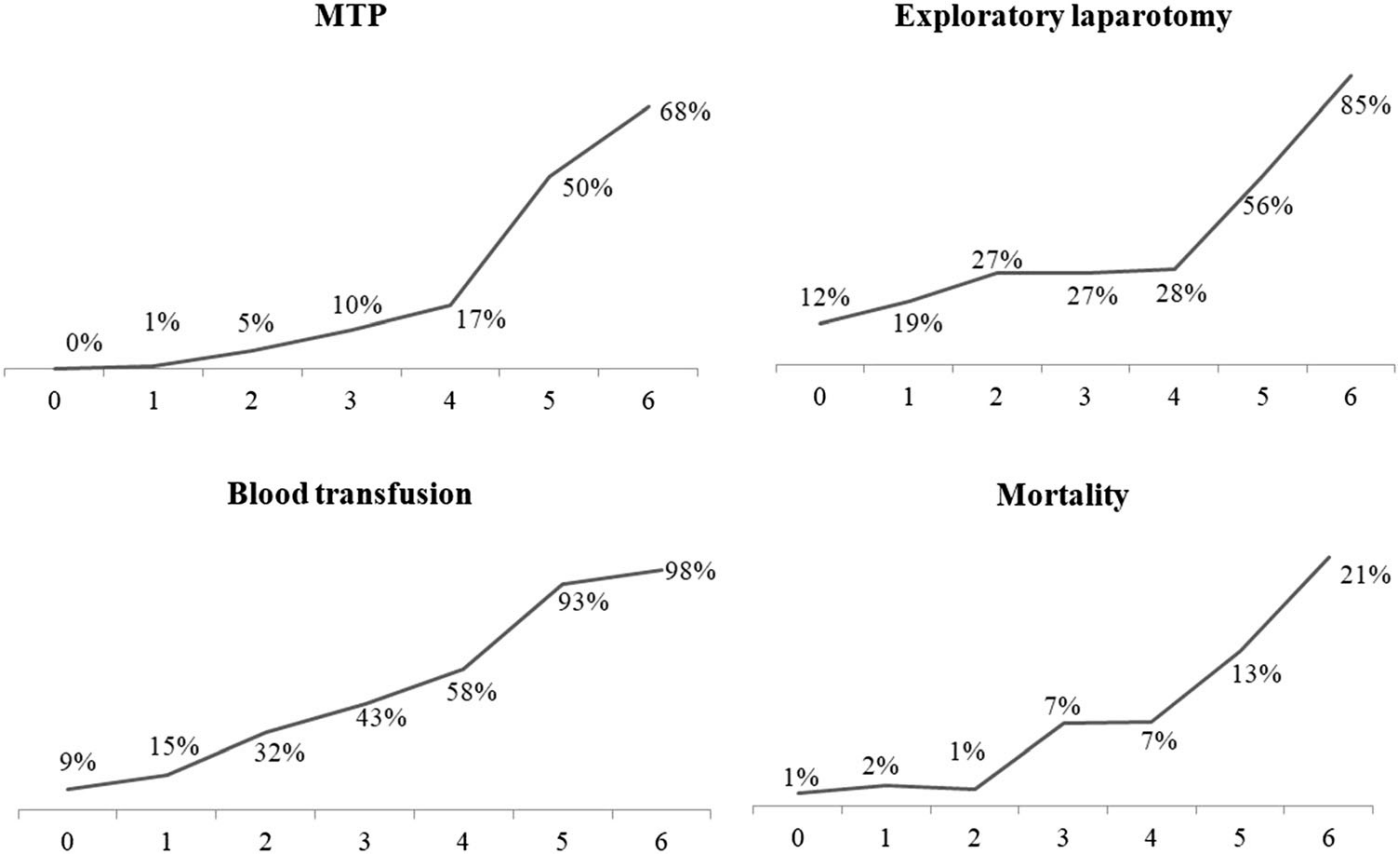
Outcomes at each FASILA scale

Figure 3 shows the association between FASILA scores and the need for exploratory laparotomy, blood transfusions, and hospital mortality. It demonstrates that the MTP activations rose from 0 to 68% with the increases in the FASILA score. From FASILA scores of 4 onwards, the proportion of exploratory laparotomies rose sharply from 24 to 85%.

The overall mortality was (*n* = 79) 6.6%, with 1/3 of deaths occurring within the first 24 h, 1/3 within the first 2 to 7 days, and around 40% occurring after 1 week of admission. Among those who died within the first 24 h, the FASILA score was 4.85 ± 1.38, compared to 4.12 ± 1.39 in those who died after 1 week. Mortality increased from 1 to 2% in patients with FASILA scores of 0 to 2 and reached 21% in patients with FASILA scores of 6 (Fig. 3).

**Fig. 4:**
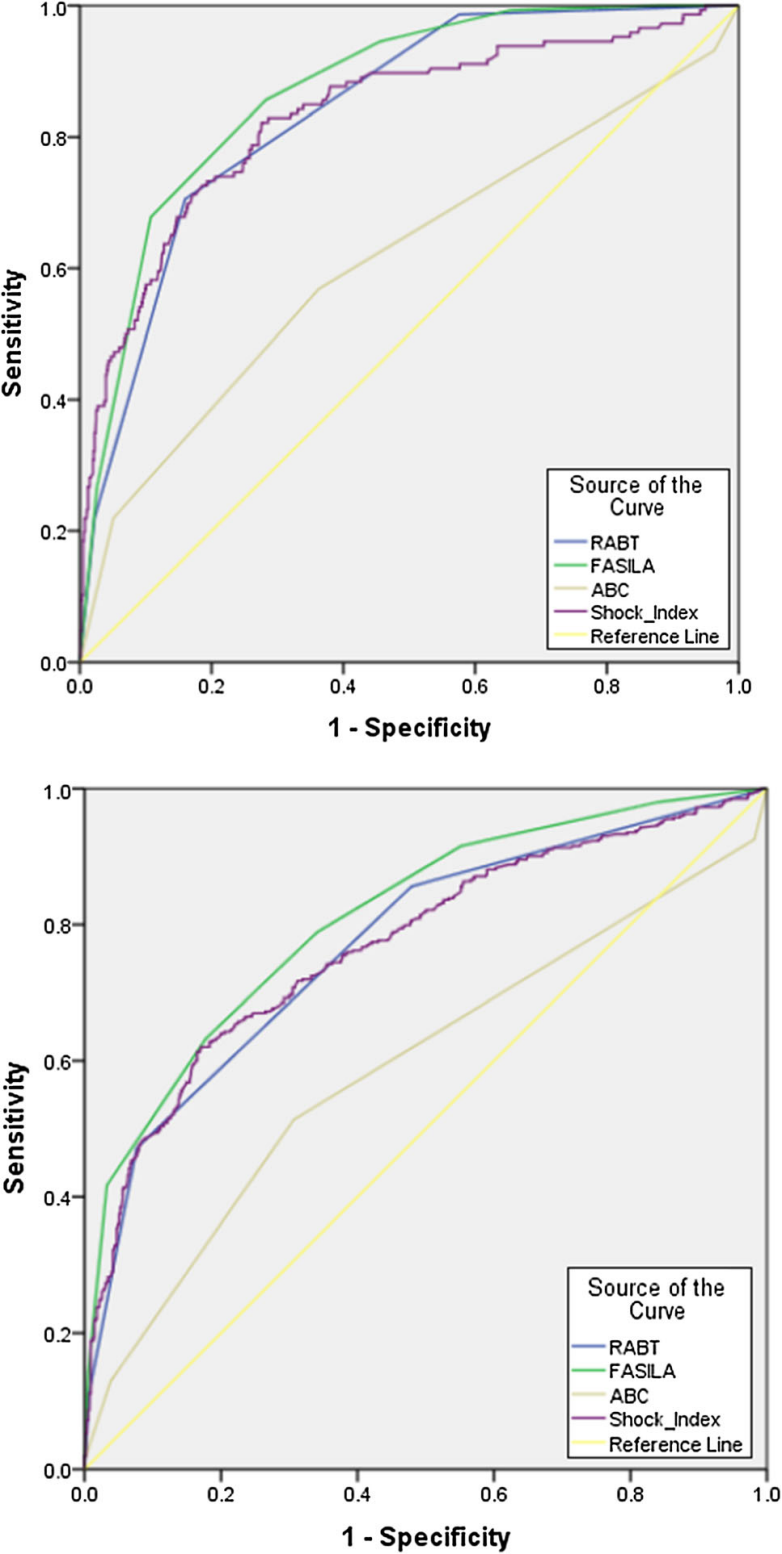
ROC curve: AUROC analysis for FASILA, RABT, ABC scores and Shock Index for MTP (upper) and blood transfusion (lower panel)

In terms of MTP, blood transfusions, and mortality, the FASILA score demonstrated a higher AUC on ROC analysis compared to the other scoring systems including RABT, ABC, and SI (Table 3 and Fig. 4). The discriminatory powers of the FASILA score in predicting MTP activation, blood transfusions, need for exploratory laparotomy, and mortality are shown in Table 3. The FASILA score had a greater NPV for mortality (96%) and MTP (94%). It had also a specificity and positive likelihood ratio of 97% and 14, respectively, for blood transfusion. Figure 4 shows the area under the receiver operating characteristics (AUROC) analysis for the FASILA, RABT, ABC, and SI in terms of MTP activation and blood transfusions. FASILA outperformed the other 3 scores.

**Table 3:** c-Statistics

	MTP*	Blood transfusion*	Mortality*	Exploratory lap*
*1 Area under the curve (AUC) for the 4 scoring systems*				
FASILA	0.87 (0.84–0.90)	0.81 (0.78–0.84)	0.77 (0.72–0.83)	0.70 (0.65–0.73)
RABT	0.84 (0.81–0.87)	0.77 (0.74–0.80)	0.64 (0.57–0.71)	0.72 (0.69–0.76)
SI	0.83 (0.79–0.87)	0.77 (0.74–0.80)	0.72 (0.66–0.79)	0.62 (0.58–0.66)
ABC score	0.61 (0.56–0.67)	0.59 (0.55–0.63)	0.51 (0.42–0.59)	0.70 (0.65–0.73)
	Blood transfusion	MT protocol	Exploratory laparotomy	Mortality
*2 Discriminatory power of FASILA score*				
Sensitivity (%)	42	68	40	55
Specificity (%)	97	89	89	83
Positive PV (%)	90	53	60	19
Negative PV (%)	69	94	78	96
+ LR	14	6.2	3.6	3.2
− LR	0.60	0.36	0.67	0.54
Accuracy (%)	73	86	75	81

*p value was significant (< 0.001) for the 4 AUCs

*SI* Shock Index, *PV* predictive value, *LR* likelihood ratio

## Discussion

The present study proposed and tested the novel FASILA score in patients sustaining abdominal injuries, for the early prediction of massive transfusion, exploratory laparotomy, and mortality. In addition to being an acronym for FAST + SI + lactate, the word FASILA also means bud or sprout (palm-cutting) in Arabic. The present study has several key findings. The FASILA score correlates well with the commonly used contemporary scores such as the RABT, ABC, and SI, in predicting blood transfusions and outcomes in trauma. However, FASILA outperforms these scores in patients with both blunt and penetrating abdominal trauma, with ease of calculation, higher AUC values, better discriminatory power, and internal validation This score reflects the current physiological and tissue perfusion status and correlates inversely with the pulse pressure, which is an important surrogate for the stroke volume. It may therefore be used subsequently as a tool to track the loss of blood volume in patients with trauma [32]. Additionally, the SI, as a component of the FASILA score, reflects the integration of the cardiovascular and sympathetic nervous systems and correlates well with the central venous oxygen saturation and early shock [33–36]. Of note, SI is directly proportional to the FASILA score in our study.

Compared to lower scores, a FASILA score of 4.5 and above was associated with a two-, three-, four-, and eightfold increase in MT, exploratory laparotomy, sepsis and mortality, and MTP activation, respectively. Moreover, a FASILA score of 4.5 doubled the duration of stay in the ICU and hospital. Compared to those who died after 1 week, 1/3 of patients who died in the first 24 h had significantly higher FASILA scores.

Ideally, predictive scores should be simple, easy to remember, reliable, efficient, and reproducible. There are many different approaches for early resuscitation of patients with trauma. This is reflected by the existence of almost 2 dozen of prediction scores for MT activation [26]. These scores share many similarities, particularly in the selection of variables used to calculate them. However, their performance is not comparable [26]. Such scores have been shown to outperform clinical judgment alone and may play a critical role in supporting the clinical decision-making [26]. Studies have shown that the SI has better predictive power than its individual components (HR, SBP) [28]. However, there is no consensus on the optimal cutoff value for SI and when it should be used. Notably, the most widely used scores such as the ABC and SI include few variables for calculation, and the components are easy to obtain in emergency settings; these factors account for their popularity [8]. The ABC score was developed and tested in patients with penetrating trauma, and it consequently has limited applicability in most patients with blunt trauma and in the elderly. In addition, it does not reflect the status of tissue perfusion on arrival. The ABC score does not rely on the SI, but on its separate components (not as a ratio). Recently, Schroll et al. [24] concluded that the SI outperformed the ABC score, being more sensitive and requiring less technical expertise in predicting the need for massive transfusion. The addition of pelvic fractures as a parameter in the SI and FAST systems increased the discriminatory power of the RABT score [28]. The RABT score has shown better sensitivity, specificity, and discriminatory power than the ABC score in predicting MT activation [28]. However, the RABT score relies on a single SI cutoff value (> 1) and lacks utility as an instant tool for indicating tissue perfusion status. Notably, the FASILA involves four different cutoff values of the SI and three ranges of lactate levels.

The SI is a good predictor of MT in various settings of trauma [34]. However, it has many limitations related to factors that affect each of its components (HR and SBP); it is also affected by the prior or concomitant use of medications and by the severity of pain [22]. Vandromme et al. [10] showed that in the pre-hospital settings, the incidence of MT rose substantially at SI levels above 0.9 in normotensive patients with blunt trauma. A study that included 8111 patients demonstrated that SI scores of 0.9 to 1.1, 1.1 to 1.3, and > 1.3 increased the risk for MT by 1.5-, 5- and eightfold, respectively [10]. Rau et al. [37] found that SI was moderately accurate in predicting the need for MT, with a cutoff value of 0.95 (AUC: 0.76). However, it had lower predictive power in patients with hypertension, diabetes, or coronary artery disease. Our previous study [22] on patients with trauma in the ED revealed that compared to SI scores of < 0.8, scores of ≥ 0.8 were associated with a higher incidence of blood transfusions (28.6 vs. 9.0%) and MT (17.7 vs. 3%) (*p* = 0.001). The cutoff value of 0.81 had a sensitivity, specificity, PPV, and NPV of 85%, 64%, 16%, and 98%, respectively [22]. In the present study, the SI scores progressed linearly with the FASILA scores; FASILA scores of > 4 were associated with SI scores of > 1 (0.97–1.46).

A recent study in patients with primary postpartum hemorrhage has shown that initial lactate levels are independently associated with the need for MT and combining lactate with SI improves the predictive performance compared to either variable alone [25]. In the search for a simple and easy predictive score for MT in cases with predominantly blunt trauma, evidence such as this led to the development of the FASILA score. In addition to other laboratory findings such as base deficit (i.e., tissue hypoperfusion and anaerobic metabolism), the initial serum lactate level is a determining factor for MT [38]. The models of both, Vandromme and the Traumatic Bleeding Severity Score (TBSS) incorporated lactate [10, 23]. Vandromme used lactate levels of ≥ 5 mmol/l as a criterion for MT along with values of SBP < 110 mm Hg, HR > 105 bpm, INR > 1.5, and Hb ≤ 11 g/dl [10]. The TBSS includes 5 variables, namely age, SBP after rapid infusion of 1000 ml of crystalloid, results of the FAST scan, severity of pelvic fractures, and lactate concentrate on arrival; the maximum TBSS value is 57 points [23]. Compared to the FASILA score, this score needs a longer time to calculate as infusions, measurements of serum lactate (routine laboratory test; not a POC test), and assessment of the severity and class of pelvic fracture requires longer time. A recent study demonstrated that pre-hospital serum lactate levels were predictive of the need for resuscitative care in normotensive patients with trauma. However, it was not better than the SI as a predictive tool [39].

FAST, a component of several scores including FASILA, is used to detect the presence of hemoperitoneum and pericardial effusion in cases of trauma. However, its accuracy is dependent on operator skills. Moreover, it cannot quantify the amount of bleeding; therefore, unless used along with other variables such as the vital signs and mode of injury, FAST has certain limitations in predicting MT. In our study, MT was given more in patients with positive FAST (35%) in comparison with 25% in patients with negative FAST, whereas in shock patients the proportion of MT was 46 to 51.5%, respectively. Rowell et al. [38] reported that FAST had a sensitivity of 62% and specificity of 83%, and therefore, in hypotensive patients with a negative FAST result, clinicians should still maintain a high index of suspicion for significant abdominal bleeding [40]. Do et al. [41] found that FAST could identify abdominal/pelvic bleeding in almost half of non-compressible torso hemorrhage (NCTH) patients, and this was not improved in patients with shock on arrival.

The parameters for the FASILA score are easy to remember; most clinicians and nurses worldwide are familiar with and use of FAST, SI, and POC serum lactate in early trauma resuscitation. The score is also easy to use and calculate, is available within few minutes of arrival, and may alert clinicians of the possibility of death in the absence of MTP activation.

### Limitations

The present study had the inherent limitations of all retrospective and single-center studies. In addition, patients with trauma who died before arrival at hospital, or presented to the ED with cardiac arrest, were not included. Assessment at admission may have been influenced by pre-hospital time and care, which included the administration of intravenous fluids and/or vasopressors. Most patients included in this analysis had blunt trauma and were adult males—less than 10% had penetrating injuries, were female, or were of the pediatric age group (< 14 years old). The present study defined MT as the transfusion of 10 or more units of blood in the first 24 h, which is beset with limitations. Many authors have attempted to overcome the limitations of this definition including Savage et al. [42] that proposed instead the critical administration threshold (CAT). Regardless of the many limitations, 10 U/24 h still remains the most utilized definition for MT worldwide. We did not include severely injured patients who die or stop bleeding with less than 10 units of RBCs, but certainly qualify. We also do not have information on the hourly transfusion.

FASILA was tested against other scoring systems but not against “clinical gestalt” alone. Pommerening et al. [43] recently demonstrated that “clinical gestalt” outperformed ABC score in predicting the need for MT in the PROMMTT study. Notably, clinical gestalt had a sensitivity of only 66%, performing poorly as a screening test for MT and missing over 1/3 of patients who ultimately required MT. In contrast, other studies have indicated the superiority of predicting scores to “clinical gestalt” in isolation, suggesting that scores such as FASILA are useful in situations where the clinical findings are equivocal or misinterpreted. While clinical findings supersede all other investigations, scoring systems such as FASILA may augment the prompt identification of ongoing hemorrhage and reduce the time to initiating life-saving hemostatic measures.

In addition, in the era of early hemostatic resuscitation, patients may require less transfusions if the need for blood products is correctly identified and immediately treated, thus increasing the value of scores such as FASILA. Lastly, confirmation of the cause of death from postmortem examinations was lacking. We plan to prospectively utilizing this score with development of a bedside app to perform this calculation on spot. Although internal validation supports the generalizability of the study, further external validation would be helpful as well.

## Conclusions

The FASILA score is a novel, simple, feasible, and easy-to-remember tool that predicts the need for blood transfusion, MTP activation, and the risk of mortality in patients with abdominal trauma. Further validation is required before widespread clinical implementation and adoption.

## Electronic supplementary material

Below is the link to the electronic supplementary material.

Supplementary file1 (DOCX 27 kb)

Supplementary file2 (DOC 84 kb)

Supplementary file3 (DOC 33 kb)

Supplementary file4 (DOC 32 kb)

## References

[1] Committee on Trauma of the American College of Surgeons (2015) ACS TQIP massive transfusion in trauma guidelines. American College of Surgeons, Chicago, IL. https://www.facs.org/~/media/files/quality%20programs/trauma/tqip/massive%20transfusion%20in%20trauma%20guildelines.ashx. Accessed 4 March 2019

[2] Callcut RA, Cripps MW, Nelson MF, Conroy AS, Robinson BB, Cohen MJ (2016). The Massive Transfusion Score as a decision aid for resuscitation: learning when to turn the massive transfusion protocol on and off. J Trauma Acute Care Surg.

[3] Callcut RA, Cotton BA, Muskat P, Fox EE, Wade CE, Holcomb JB, Schreiber MA, Rahbar MH, Cohen MJ, Kundson MM (2013). Defining when to initiate massive transfusion: a validation study of individual massive transfusion triggers in PROMMTT patients. J Trauma Acute Care Surg.

[4] Callcut RA, Johannigman JA, Kadon KS, Hanseman DJ, Robinson BR (2011). All massive transfusion criteria are not created equal: defining the predictive value of individual transfusion triggers to better determine who benefits from blood. J Trauma.

[5] Dente CJ, Shaz BH, Nicholas JM, Harris RS, Wyrzykowski AD, Patel S, Shah A, Vercruysee GA, Feliciano DV, Rozycki GS (2009). Improvements in early mortality and coagulopathy are sustained better in patients with blunt trauma after institution of a massive transfusion protocol in a civilian level I trauma center. J Trauma.

[6] del Junco DJ, Holcomb JB, Fox EE, Brasel KJ, Phelan HA, Bulger EM, Schreiber MA, Muskat P, Alarcon LH, Cohen MJ (2013). Resuscitate early with plasma and platelets or balance blood products gradually: findings from the PROMMTT study. J Trauma Acute Care Surg.

[7] Yucel N, Lefering R, Maegele M, Vorweg M, Tjardes T, Ruchholtz S, Neugebauer EA, Wappler F, Bouillon B, Rixen D, Polytrauma Study Group of the German Trauma Society (2006). Trauma Associated Severe Hemorrhage (TASH)-Score: probability of mass transfusion as surrogate for life threatening hemorrhage after multiple trauma. J Trauma.

[8] Nunez TC, Voskresensky IV, Dossett LA, Shinall R, Dutton WD, Cotton BA (2009). Early prediction of massive transfusion in trauma: simple as ABC (assessment of blood consumption)?. J Trauma.

[9] Rainer TH, Ho AM, Yeung JH, Cheung NK, Wong RS, Tang N, Ng SK, Wong GK, Lai PB, Graham CA (2011). Early risk stratification of patients with major trauma requiring massive blood transfusion. Resuscitation.

[10] Vandromme MJ, Griffin RL, McGwin G, Weinberg JA, Rue LW, Kerby JD (2011). Prospective identification of patients at risk for massive transfusion: an imprecise endeavor. Am Surg.

[11] McLaughlin DF, Niles SE, Salinas J (2008). A predictive model for massive transfusion in combat casualty patients. J Trauma.

[12] Schreiber MA, Perkins J, Kiraly L (2007). Early predictors of massive transfusion in combat casualties. J Am Coll Surg.

[13] Cancio LC, Wade CE, West SA, Holcomb JB (2008). Prediction of mortality and of the need for massive transfusion in casualties arriving at combat support hospitals in Iraq. J Trauma.

[14] Wade CE, Holcomb JB, Chrisholm GB, Michalek JE (2008) Accurate and early prediction of massive transfusion in trauma patients. In: 67th Annual meeting of the American Association for the Surgery in Trauma, Maui (Hawaii)

[15] Moore F, McKinley B, Moore E, Nathens A, Rhee P, Puyana J, Beilman G, Cohn S (2007). Need for massive transfusion can be predicted early after trauma center arrival. J Trauma..

[16] Baker JB, Korn CS, Robinson K, Chan L, Henderson SO (2001). Type and crossmatch of the trauma patient. J Trauma.

[17] Ruchholtz S, Pehle B, Lewan U, Lefering R, Müller N, Oberbeck R, Waydhas C (2006). The emergency room transfusion score (ETS): prediction of blood transfusion requirement in initial resuscitation after sever trauma. Transfus Med.

[18] Cotton BA, Faz G, Hatch QM, Radwan ZA, Podbielski J, Wade C, Kozar RA, Holcomb JB (2011). Rapid thrombelastography delivers real-time results that predict transfusion within 1 hour of admission. J Trauma.

[19] Davenport R, Manson J, De'Ath H, Platton S, Coates A, Allard S, Hart D, Pearse R, Pasi KJ, MacCallum P, Stanworth S, Brohi K (2011). Functional definition and characterization of acute traumatic coagulopathy. Crit Care Med.

[20] Larson CR, White CE, Spinella PC, Jones JA, Holcomb JB, Blackbourne LH, Wade CE (2010). Association of shock, coagulopathy, and initial vital signs with massive transfusion in combat casualties. J Trauma.

[21] Eastridge BJ, Butler F, Wade CE, Holcomb JB, Salinas J, Champion HR, Blackbourne LH (2010). Field triage score (FTS) in battlefield casualties: validation of a novel triage technique in a combat environment. Am J Surg.

[22] El-Menyar A, Goyal P, Tilley E, Latifi R (2018). The clinical utility of shock index to predict the need for blood transfusion and outcomes in trauma. J Surg Res.

[23] Ogura T, Nakamura Y, Nakano M, Izawa Y, Nakamura M, Fujizuka K, Lefor AT (2014). Predicting the need for massive transfusion in trauma patients: the traumatic bleeding severity score. J Trauma Acute Care Surg.

[24] Schroll R, Swift D, Tatum D, Couch S, Heaney JB, Llado-Farrulla M, Zucker S, Gill F, Brown G, Buffin N, Duchesne J (2018). Accuracy of shock index versus ABC scoreto predict need for massive transfusion in trauma patients. Injury.

[25] Sohn CH, Kim YJ, Seo DW, Won HS, Shim JY, Lim KS, Kim WY (2018). Blood lactate concentration and shock index associated with massive transfusion in emergency department patients with primary postpartum haemorrhage. Br J Anaesth.

[26] El-Menyar A, Mekkodathil A, Abdelrahman H, Latifi R, Galwankar S, Al-Thani H, Rizoli S (2019). Review of existing scoring systems for massive blood transfusion in trauma patients: where do we stand?. Shock.

[27] https://www.radiometer.com/en/products/blood-gas-testing/abl90-flex-blood-gas-analyzer. Accessed 1 June 2019

[28] Joseph B, Khan M, Truitt M, Jehan F, Kulvatunyou N, Azim A, Jain A, Zeeshan M, Tang A, O'Keeffe T (2018). Massive transfusion: the revised assessment of Bleeding and Transfusion (RABT) Score. World J Surg.

[29] Singh J, Gupta G, Garg R, Gupta A (2011). Evaluation of trauma and prediction of outcome using TRISS method. J Emerg Trauma Shock.

[30] Arumugam S, Al-Hassani A, El-Menyar A, Abdelrahman H, Parchani A, Peralta R, Zarour A, Al-Thani H (2015). Frequency, causes and pattern of abdominal trauma: a 4-yeardescriptive analysis. J Emerg Trauma Shock.

[31] Steyerberg EW, Harrell FE, Borsboom GJ, Vegouwe Y, Habbema JD (2001). Internal validation of predictive models: efficiency of some procedures for logistic regression analysis. J Clin Epidemiol.

[32] Convertino VA, Cooke WH, Holcomb JB (2006). Arterial pulse pressure and its association with reduced stroke volume during progressive central hypovolemia. J Trauma.

[33] Tongtong Yu, Tian C, Song J, He D, Sun Z, Sun Z (2017). Derivation and validation of shock index as a parameter for predicting long-term prognosis in patients with acute coronary syndrome. Sci Rep.

[34] DeMuro JP, Simmons S, Jax J, Gianelli SM (2013). Application of the shock index to the prediction of need for hemostasis intervention. Am J Emerg Med.

[35] El-Menyar A, Jabbour G, Asim M, Abdelrahman H, Mahmood I, Al-Thani H (2019). Shock index in patients with traumatic solid organ injury as a predictor of massive blood transfusion protocol activation. Inj Epidemiol.

[36] El-Menyar A, Sulaiman K, Almahmeed W, Al-Motarreb A, Asaad N, AlHabib KF, Alsheikh-Ali AA, Al-Jarallah M, Singh R, Yacoub M, Al SJ (2019). Shock index in patients presenting with acute heart failure: a multicenter multinational observational study. Angiology.

[37] Rau CS, Wu SC, Kuo SC, Pao-Jen K, Shiun-Yuan H, Chen YC, Hsieh HY, Hsieh CH, Liu HT (2016). Prediction of massive transfusion in trauma patients with shock index, modified shock index, and age shock index. Int J Environ Res Public Health.

[38] Dente CJ, Shaz BH, Nicholas JM, Harris RS, Wyrzykowski AD, Ficke BW, Vercruysse GA, Feliciano DV, Rozycki GS, Salomone JP, Ingram WL (2010). Early predictors of massive transfusion in patients sustaining torso gunshot wounds in a civilian level I trauma center. J Trauma..

[39] St John AE, McCoy AM, Moyes AG, Guyette FX, Bulger EM, Sayre MR (2018). Prehospital lactate predicts need for resuscitative care in non-hypotensive trauma patients. West J Emerg Med.

[40] Rowell SE, Barbosa RR, Holcomb JB, Fox EE, Barton CA, Schreiber MA (2019). The focused assessment with sonography in trauma (FAST) in hypotensive injured patients frequently fails to identify the need for laparotomy: a multi-institutional pragmatic study. Trauma Surg Acute Care Open.

[41] Do WS, Chang R, Fox EE, Wade CE, Holcomb JB, Martin MJ, NCTH Study Group (2019). Too fast, or not fast enough? The FAST exam in patients with non-compressible torso hemorrhage. Am J Surg.

[42] Savage SA, Sumislawski JJ, Zarzaur BL, Dutton WP, Croce MA, Fabian TC (2015). The new metric to define large-volume hemorrhage: results of a prospective study of the critical administration threshold. J Trauma Acute Care Surg.

[43] Pommerening MJ, Goodman MD, Holcomb JB, Wade CE, Fox EE, Del Junco DJ, Brasel KJ, Bulger EM, Cohen MJ, Alarcon LH, Schreiber MA, Myers JG, Phelan HA, Muskat P, Rahbar M, Cotton BA, MPH on behalf of the PROMMTT Study Group (2015). Clinical gestalt and the prediction of massive transfusion after trauma. Injury.

