# Supplementary material for: The FASILA Score: A Novel Bio-Clinical Score to Predict Massive Blood Transfusion in Patients with Abdominal Trauma
Source: World J Surg. 2019 Nov 20;44(4):1126–36. doi: 10.1007/s00268-019-05289-0 (PMC7223809; doi:10.1007/s00268-019-05289-0)

Suppl Fig. 1: Receiver operating characteristics (ROC) and areas under the curves (AUC).for optimum FASILA cutoff
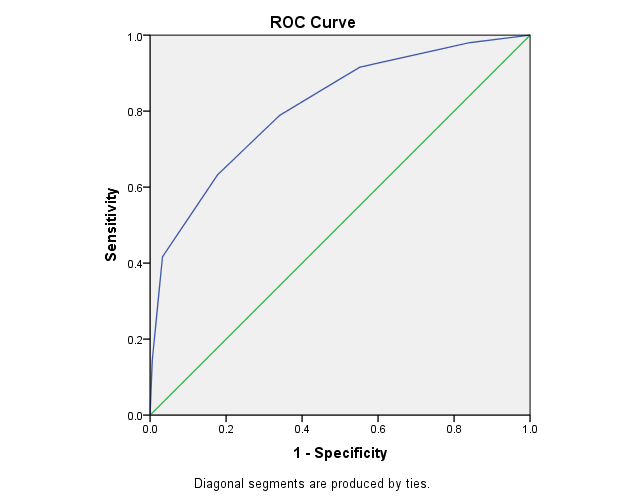

Supplement: Supplementary file 1 — Supplementary file1 (DOCX 27 kb) [file 268_2019_5289_MOESM1_ESM.docx]
