# Supplementary material for: The FASILA Score: A Novel Bio-Clinical Score to Predict Massive Blood Transfusion in Patients with Abdominal Trauma
Source: World J Surg. 2019 Nov 20;44(4):1126–36. doi: 10.1007/s00268-019-05289-0 (PMC7223809; doi:10.1007/s00268-019-05289-0)
Supplement: Supplementary file 3 — Supplementary file3 (DOC 33 kb) [file 268_2019_5289_MOESM3_ESM.doc]

| **Suppl Table 2: comparison between users of massive transfusion (MT) versus no-massive transfusion** | | | |
| --- | --- | --- | --- |
|  | **Not MT(<10 unit)** | **MT(≥10 unit)** | **P** |
| ISS | 22(4-59) | 33(9-66) | 0.001 |
| TRISS | 0.985(0.101-0.999) | 0.916(0.059-0.999) | 0.001 |
| RTS | 8(3-9) | 7(1.5-9) | 0.001 |
| FASILA | 4(0-6) | 5(1-6) | 0.001 |
| RABT | 2(1-4) | 3(1-4) | 0.001 |
| ABC | 2.5(1-5) | 3(1-4) | 0.76 |
| Shock index | 0.88(0.45-3.46) | 1.2(0.43-3.25) | 0.001 |
| Exploratory laparotomy | 38% | 64.5% | 0.001 |
| Ventilatory days | 4(1-40) | 11(1-73) | 0.001 |
| ICU days | 5(1-81) | 15(1-161) | 0.001 |
| Hospital length of stay | 16(1-138) | 32(1-505) | 0.001 |
| Mortality | 8.8% | 32.6% | 0.001 |
