# Supplementary material for: The FASILA Score: A Novel Bio-Clinical Score to Predict Massive Blood Transfusion in Patients with Abdominal Trauma
Source: World J Surg. 2019 Nov 20;44(4):1126–36. doi: 10.1007/s00268-019-05289-0 (PMC7223809; doi:10.1007/s00268-019-05289-0)
Supplement: Supplementary file 4 — Supplementary file4 (DOC 32 kb) [file 268_2019_5289_MOESM4_ESM.doc]

| Suppl Table 3: FASILA lower versus higher than the optimum cut-off score | | | |
| --- | --- | --- | --- |
|  | FASILA <4.5 (81.3%) | FASILA ≥4.5 (18.7%) | P |
| ISS | 14(1-59) | 27(1-66) | 0.001 |
| RTS | 8(2-8) | 7(3-8) | 0.001 |
| TRISS | 0.993(0.087-0.999) | 0.972(0.115-0.999) | 0.001 |
| Exploratory laparotomy | 21.4% | 59.7% | 0.001 |
| MTP | 6.1% | 53.2% | 0.001 |
| MT | 20.4% | 43.5% | 0.001 |
| sepsis | 1.8% | 9.1% | 0.001 |
| ICU days | 4(1-81) | 7(1-161) | 0.001 |
| Hospital length of stay | 7(1-505) | 19(1-193) | 0.001 |
| Mortality | 3.8% | 18.8% | 0.001 |
